# Spontaneous iliopsoas muscle hematoma in a patient with von Willebrand disease: a case report

**DOI:** 10.1186/1752-1947-5-274

**Published:** 2011-07-02

**Authors:** Bijan Keikhaei, Ahmad Soltani Shirazi

**Affiliations:** 1 Department of Thalassemia & Hemoglobinopathy Research Center, Ahvaz Jundishapur University of Medical Science, Shafa Hospital (Golestan Area), Ahvaz 61357-33119, Iran; 2 Department of Radiology, Ahvaz Jundishapur University of Medical Science, Golestan Hospital (Golestan Area), Ahvaz 61357-33118, Iran

## Abstract

**Introduction:**

Iliopsoas hemorrhage is a serious complication of bleeding disorders that occurs most commonly in patients with hemophilia and less commonly in patients with von Willebrand disease. It causes severe pain, muscle dysfunction and occasionally femoral nerve palsy. We describe the case of a patient with von Willebrand disease type 3 with a large iliopsoas hematoma who was treated with a von Willebrand factor concentrate (Humate-P).

**Case presentation:**

A 20-year-old Iranian man was referred to our emergency ward because of the gradual onset of right flank pain. He was known to have been diagnosed with von Willebrand disease type 3 at age two years old. Magnetic resonance imaging showed a mass in the right iliopsoas muscle. The diagnosis of iliopsoas hemorrhage and partial femoral nerve palsy was established, and he responded to medical treatment.

**Conclusion:**

We report a case of von Willebrand disease type 3 with spontaneous iliopsoas hematoma associated with femoral nerve palsy that was well managed with Humate-P treatment.

## Introduction

Von Willebrand disease (vWD) is a hereditary bleeding disorder caused by a quantitative or qualitative defect of von Willebrand factor. The prevalence of this disease is estimated to be 1% to 2% of the general population. Von Willebrand factor is a coagulation factor for initial stages of blood clotting and is also a carrier for factor VIII. Patients with vWD develop coagulation platelet plugs and red coagulation clots are unable to form. vWD consists of a broad variation of clinical manifestations, with one extreme being a minimal bleeding tendency and the other being a severe presentation such as severe hemophilia A. Luckily, most of these patients present with mild to moderate clinical symptoms. The severe type of the vWD, type 3, occurs in less than 5% of patients. The inheritance pattern of this type is usually autosomal recessive. Patients with vWD usually show clinical features such as recurrent nosebleeds, easy bruising, bleeding post-tooth extraction, post-tonsillectomy or other surgical bleeding, and excessive menstrual bleeding.

Musculoskeletal hemorrhage occurs commonly in vWD type 3-like hemophilia A. Iliopsoas hemorrhage is a potentially serious complication because a delay in beginning treatment may result in permanent femoral nerve palsy [1]. The present case report describes a man with vWD type 3 who had a spontaneous iliopsoas hematoma that responded well to Humate-P treatment (CSL Behring, King of Prussia, PA, USA) [2].

## Case presentation

**Figure 1:**
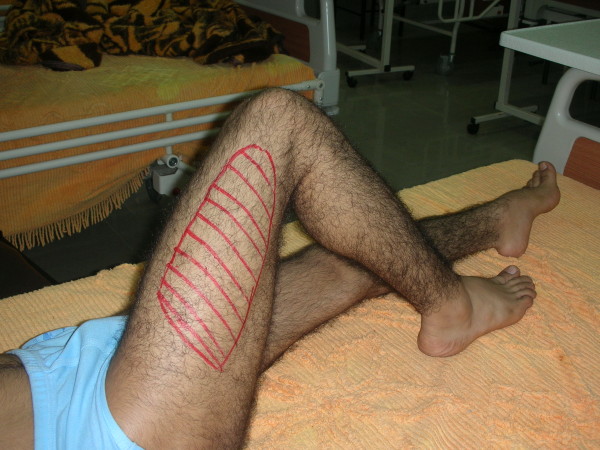
**Anti-pain posture showing marked hypoesthesia area**.

**Figure 2:**
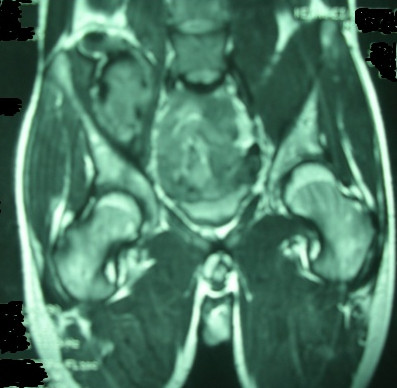
MRI scan shows a large hyper signal mass in the right iliopsoas muscle

A 20-year-old Iranian man came to our hospital because of the gradual onset of right flank pain. He was known to have had vWD type 3 since he was two years old. His laboratory findings were as follows: B-positive blood type, partial thromboplastin time 56 seconds, bleeding time more than 12 minutes, hemoglobin 10 g/dL, mean corpuscular volume 75 fL, mean corpuscular hemoglobin 24 pg/red blood cell, factor VIIIc activity 3%, vWF antigen level was undetectable, and vWF ristocetin cofactor activity was undetectable. He had experienced some occasional hemarthrosis, but he had been well for two months before he presented to our hospital. He had no history of trauma. However, his physical examination showed that he had an anxious and painful facial expression. There was severe tenderness on palpation of the right flank. No mass was found. His pain radiated to the hip joint and the inguinal region. He supported himself with a stick and could not stand up on his legs. The patient exhibited an anti-pain posture and held his right leg flexed at the hip joint (Figure 1). He had hypoesthesia on the anterior aspect of his right thigh (Figure 1). His knee tendon reflex was weak, and weakness of his quadriceps muscle was demonstrable. A plain abdominal X-ray demonstrated lack of clarity at the right psoas muscle line. Magnetic resonance imaging (MRI) showed a hematoma in the right iliopsoas muscle (Figure 2). He was treated with Humate-P at a dose of 40 IU/kg body weight twice daily. The dose was calculated on the basis of factor VIII coagulation. He was treated with the Humate-P regimen for three days, after which his symptoms resolved completely and he resumed walking without support. The treatment continued for the next three days. At present, he has been well for three months and has not experienced any further symptoms or complications. His follow-up physical examination showed a healthy quadriceps muscle function, a good patellar tendon reflex and an intact sensory femoral nerve.

## Discussion

Iliopsoas hemorrhage, whether spontaneous or traumatic, is encountered in a variety of coagulation disorders, such as hemophilia and vWD, and in association with anti-coagulant drug treatments such as heparin, warfarin and ticlopidine; however, most cases of iliopsoas hemorrhage occur in patients with hemophilia [3], and the first case was reported by Tallroth in 1939 [4].

The diagnosis of retroperitoneal hematoma is made on the basis of ultrasonography, computed tomography and MRI. The treatment of patients with iliopsoas muscle hemorrhage remains controversial. Some experts consider that surgical decompression (open or percutaneous aspiration) is the treatment of choice because it minimizes neurological deficits. Others have advocated a medical approach. Non-operative treatment consists of complete rest and replacement factor therapy. Iliopsoas hemorrhage occurs mostly in patients with hemophilia after trauma (falling from height), but our patient had no history of trauma. Good results were achieved by administering Humate-P treatment to our patient [5].

## Conclusion

This report describes the case of a patient with vWD type 3 who had a spontaneous iliopsoas hematoma. The diagnosis was established on the basis of MRI, and the patient showed a good response to Humate-P treatment. We could not find any similar case reported in the medical literature, and we think that our case report may be the first of its kind to be published. The publication of this article may raise the clinician's awareness of the treatment of iliopsoas hematoma in patients with vWD.

## Competing interests

The authors declare that they have no competing interests.

## Consent

Written informed consent was obtained from the patient for publication of this case report and any accompanying images. A copy of the written consent is available for review by the Editor-in-Chief of this journal.

## Authors' contributions

The article was written by BK, and the imaging studies were interpreted by ASS. Both authors have read and approved the final manuscript.
